# Impact of different parametric Patlak imaging approaches and comparison with a 2-tissue compartment pharmacokinetic model with a long axial field-of-view (LAFOV) PET/CT in oncological patients

**DOI:** 10.1007/s00259-024-06879-4

**Published:** 2024-09-11

**Authors:** Leyun Pan, Christos Sachpekidis, Jessica Hassel, Petros Christopoulos, Antonia Dimitrakopoulou-Strauss

**Affiliations:** 1 Clinical Cooperation Unit Nuclear Medicine, German Cancer Research Center (DKFZ), Im Neuenheimer Feld 280, D-69210 Heidelberg, Germany; 2 Department of Dermatology and National Center for Tumor Diseases (NCT), University Hospital Heidelberg, Heidelberg, Germany; 3 Department of Thoracic Oncology, Thoraxklinik of the University of Heidelberg, Heidelberg, Germany

**Keywords:** Long axial field-of-view PET/CT, [^18^F]FDG PET/CT, Kinetic modelling, Patlak parametric imaging, 2-tissue compartment model

## Abstract

**Aim:**

The recently introduced Long-Axial-Field-of-View (LAFOV) PET-CT scanners allow for the first-time whole-body dynamic- and parametric imaging. Primary aim of this study was the comparison of direct and indirect Patlak imaging as well as the comparison of different time frames for Patlak calculation with the LAFOV PET-CT in oncological patients. Secondary aims of the study were lesion detectability and comparison of Patlak analysis with a two-tissue-compartment model (2TCM).

**Methodology:**

50 oncological patients with 346 tumor lesions were enrolled in the study. All patients underwent [^18^F]FDG PET/CT (skull to upper thigh). Here, the Image-Derived-Input-Function) (IDIF) from the descending aorta was used as the exclusive input function. Four sets of images have been reviewed visually and evaluated quantitatively using the target-to-background (TBR) and contrast-to-noise ratio (CNR): short-time (30 min)-direct (STD) Patlak K_i_, short-time (30 min)-indirect (STI) Patlak K_i_, long-time (59.25 min)-indirect (LTI) Patlak K_i_, and 50–60 min SUV (sumSUV). VOI-based 2TCM was used for the evaluation of tumor lesions and normal tissues and compared with the results of Patlak model.

**Results:**

No significant differences were observed between the four approaches regarding the number of tumor lesions. However, we found three discordant results: a true positive liver lesion in all Patlak K_i_ images, a false positive liver lesion delineated only in LTI K_i_ which was a hemangioma according to MRI and a true negative example in a patient with an atelectasis next to a lung tumor. STD, STI and LTI K_i_ images had superior TBR in comparison with sumSUV images (2.9-, 3.3- and 4.3-fold higher respectively). TBR of LTI K_i_ were significantly higher than STD K_i_. VOI-based k_3_ showed a 21-fold higher TBR than sumSUV. Parameters of different models vary in their differential capability between tumor lesions and normal tissue like Patlak K_i_ which was better in normal lung and 2TCM k_3_ which was better in normal liver. 2TCM K_i_ revealed the highest correlation (*r* = 0.95) with the LTI Patlak K_i_ in tumor lesions group and demonstrated the highest correlation with the STD Patlak K_i_ in all tissues group and normal tissues group (*r* = 0.93 and *r* = 0.74 respectively).

**Conclusions:**

Dynamic [^18^F]-FDG with the new LAFOV PET/CT scanner produces Patlak K_i_ images with better lesion contrast than SUV images, but does not increase the lesion detection rate. The time window used for Patlak imaging plays a more important role than the direct or indirect method. A combination of different models, like Patlak and 2TCM may be helpful in parametric imaging to obtain the best TBR in the whole body in future.

**Supplementary Information:**

The online version contains supplementary material available at 10.1007/s00259-024-06879-4.

## Introduction

The introduction of Long-Axial-Field-of-View (LAFOV) PET-CT scanners has marked a new era in molecular imaging. Whole body PET-CT studies can be performed with lower doses of a radiopharmaceutical and within a short acquisition time at least for routine purposes. All these technological improvements have a major impact particularly in oncological patients. Furthermore, LAFOV PET-CT systems allow for the first-time whole body (WB) dynamic imaging and WB pharmacokinetic studies [1–3]. This is in particular interesting for the evaluation of novel radiopharmaceuticals. The potential of WB dynamic imaging including pharmacokinetic modeling and parametric imaging for the most common used radiopharmaceutical, 2-deoxy-2- [fluorine-18] fluoro-D-glucose ([^18^F]FDG), is under evaluation. [^18^F]FDG is still used in the clinical routine of oncology for several indications, including diagnosis, staging, restaging as well therapy response evaluation [4].

In most oncological studies, the acquisition protocol includes the skull base to the upper thigh, which covers most relevant portions of the body. The recently introduced LAFOV PET/CT systems, such as the Biograph Vision Quadra (Siemens Healthineers) with approximately one meter FOV and the total body uEXPLORER (United Imaging) with approximately two meters FOV, which are digital total-body PET/CT systems enable both the coverage of one to two meter within one position and a significant increase in system sensitivity [1, 3, 5, 6]. In dynamic PET in particular, the new scanners dramatically enhance its capabilities, enabling for the first time the dynamic acquisition of the body trunk in a single measurement. This allows the simultaneous evaluation of radiotracer kinetics of most organs and tumor lesions, using large vessels for image-derived input function (IDIF) calculation, thus providing robust information on in vivo tracer biology [7–9].

The dynamic study of the new scanner also produces a much larger dataset than the traditional PET/CT scanner. The feasibility of kinetic modelling methods like two-tissue-compartment model (2TCM) and Patlak model also need to be evaluated with the new LAFOV system [10, 11]. In addition to volume-of-interest (VOI)-based analyses using the averaged time activity curve (TAC) from a VOI, kinetic modelling can generate parametric images of isolated parameters of the radiotracer pharmacokinetics at the voxel level. Parametric images can be generated from reconstructed dynamic PET images, known as indirect method, or directly from PET sinogram data, known as direct method [12].

In the present study, we investigated oncological patients with the new LAFOV Biograph Vision Quadra PET/CT after application of low-dose [^18^F] FDG. Our aim was to evaluate whether Patlak imaging is feasible with a new LAFOV system. The primary aim of this study was the comparison of direct and indirect Patlak imaging as well as the comparison of different time frames for Patlak calculation with the LAFOV PET-CT in oncological patients. Secondary objectives of the study were lesion detectability and comparison of Patlak analysis with a VOI-based two-tissue-compartment model (2TCM) analysis.

## Materials and methods

### Patients

A total of 50 consecutive oncological patients with different tumor entities (mean age 63.4 years, range 21–91 years) were enrolled in this retrospective analysis of prospectively designed study protocols and underwent dynamic [^18^F] FDG PET/CT for staging or re-staging purposes or as baseline study prior to onset to treatment. All patients had histologically confirmed tumors. Patient characteristics are summarized in Table 1. The study was conducted in accordance with the Declaration of Helsinki, and was approved by the Ethics Committee of the University of Heidelberg (S-107/2012, S-879/2020, S-950/2021). All patients gave written informed consent to undergo [^18^F]FDG and to have their medical records released. Patient preparation was done according to the EANM guidelines for tumor imaging [13].

**Table 1:** Characteristics of the patients investigated

Patient Nr.	Gender	Age	Inj.Dose (MBq)	Tumor	Patient Nr.	Gender	Age	Inj.Dose (MBq)	Tumor
1	F	91	125	Melanoma	26	F	76	93.48	Melanoma
2	F	62	171	Melanoma	27	M	64	140.04	Melanoma
3	M	60	192	Melanoma	28	M	66	179.63	Melanoma
4	F	73	182	Non-small cell lung cancer	29	M	37	158.8	Uvea Melanoma
5	M	83	251	Melanoma	30	F	40	270.78	Sarcoma*
6	M	72	153.12	Small cell lung cancer	31	M	83	183.44	Melanoma
7	M	21	176.69	Sarcoma	32	M	60	138.27	Melanoma
8	M	66	176	Melanoma	33	M	65	159.77	Bladder tumor
9	F	70	107	Melanoma	34	F	60	121.22	Melanoma
10	M	79	198	Melanoma	35	F	69	138.62	Non-small cell lung cancer
11	M	47	229	Melanoma	36	M	75	173.48	Uvea Melanoma
12	F	59	121.	Non-small cell lung cancer	37	F	60	165.68	Non-small cell lung cancer
13	M	69	243.13	Chondrosarcoma*	38	M	60	165.14	Non-small cell lung cancer
14	F	61	117.69	Small cell lung cancer	39	M	57	132.65	Non-small cell lung cancer
15	M	68	199.59	Uvea Melanoma	40	F	64	142.46	Non-small cell lung cancer
16	M	41	139.68	Melanoma	41	M	61	139.15	Non-small cell lung cancer
17	M	75	146.22	Melanoma	42	M	66	170.21	Small cell lung cancer
18	F	41	224.15	Sarcoma	43	M	64	166.54	Pleural mesothelioma
19	M	65	152.4	Melanoma	44	M	56	176.34	Melanoma
20	F	23	175.55	Sarcoma	45	M	64	143.35	Small cell lung cancer
21	F	72	117.39	Melanoma	46	M	78	162.59	Small cell lung cancer
22	M	80	171.82	Melanoma	47	F	73	129.01	Small cell lung cancer
23	M	67	155.52	Lymphoma	48	M	74	158.19	Bladder tumor
24	F	62	169.87	Uvea Melanoma	49	F	65	113.31	Non-small cell lung cancer
25	F	60	94.81	Non-small cell lung cancer	50	F	66	102.02	Uvea Melanoma

*patients without metastases

### PET/CT examination

All patients fasted for at least 6 h before [^18^F]FDG administration. Patients underwent PET/CT with a LAFOV scanner (Biograph Vision Quadra, Siemens Co., Erlangen, Germany) after intravenous administration of a body weight-adjusted activity of 2 MBq/kg [^18^F]-FDG (mean 160 MBq; range 102–270 MBq).

PET/CT dynamic data acquisition was performed from the top of the head to the upper thigh (FOV 106 cm) for 60 min after i.v. injection of the radiotracer using a 33-frame protocol (10 frames of 15 s, 5 frames of 30 s, 5 frames of 60 s, 5 frames of 120 s, and 8 frames of 300 s).

All PET images were acquired in high resolution mode (HS mode, 18° acceptance angle), attenuation-corrected and an image matrix of 440 × 440 pixels was used for iterative image reconstruction. Images were reconstructed using the manufacturer’s standard reconstruction method (Siemens Healthineers) using the point spread function + time-of-flight algorithm (PSF + TOF, 4 iterations x 5 subsets) without Gaussian filtering into 1.65 × 1.65 × 1.65 mm^3^ voxels. A low-dose attenuation CT (120 kV, 30 eff. mA) was used for attenuation correction of the dynamic emission PET data and for image fusion.

### Patlak imaging

Whole-body parametric images (FOV: 106 cm) were generated using the direct and indirect Patlak methods, with the Image-Derived Input Function (IDIF) of the descending aorta as the exclusive input function. In the direct Patlak reconstruction method, we used a dedicated Patlak module implemented in the e7 tools that is an investigational research prototype software for PET image reconstruction and parametric imaging (Siemens Healthineers). Following the recommendation from the experts of Siemens Healthineers, we only used the short-time 30 min protocol in 6 frames (last 6 frames of 300 s), which is referenced as short-time-direct (STD) protocol. In the indirect Patlak reconstruction method, we used a dedicated software PMOD (PMOD Technologies, Zurich, Switzerland), which can setup time protocol fast and easily. We tested two different time protocols: (1) the same protocol as the one used for direct Patlak which is referenced as short-time-indirect (STI) protocol; (2) A long-time 59.25 min protocol consisted of 30 frames, which skipped the first 3 frames in order to reduce the background of blood vessels and is referenced as long-time-indirect (LTI) protocol.

### Data analysis

#### Visual assessment of Patlak parametric images

Patlak parametric image analysis was performed using a dedicated imaging workstation and software (aycan Osirix^PRO^). Two experienced, board-certified nuclear medicine physicians well versed in PET oncological diagnosis and pharmacokinetic modeling (CS, ADS) read the datasets together and any disagreements were resolved by consensus.

Visual analysis was based on the identification of sites of focally enhanced [^18^F]FDG uptake relative to local background, which were considered suggestive of tumor involvement (tumor lesions) after disregarding known benign [^18^F]FDG avid structures, such as sites of unspecific uptake after comparison with the low dose CT and the patient history, e.g. immune-related adverse events (irAEs) in melanoma patients after immunotherapy or pneumonitis in lung cancer patients etc. The number of tumor lesions was determined in each scan, with a maximum of up to 20 lesions being calculated per patient. With regard to lesion detectability, the results of the 10-min SUV images served as a reference for the comparison with the results of the Patlak K_i_ images (STD, STI and LTI). Reference for the tumor lesions was the clinical follow-up and other diagnostic imaging modalities, like diagnostic CT and MRI. The majority of the patients (48/50) had a metastatic disease and received therefore oncological treatment. A histological confirmation of every metastasis was not possible.

#### Objective evaluation of Patlak parametric image quality

Evaluation of the dynamic PET/CT data was also based on VOIs drawn over tumor lesions and normal tissues. Normal tissues included the following organs: liver, kidney, lung, spleen, bone and muscle. In particular, tumor lesions were assessed using irregular VOIs using an isocontour mode and placed over the entire lesions. For normal organs in the liver, spleen and lung, VOIs were drawn after placing spherical VOIs covering approximately five consecutive slices and using an isocontour mode. For the kidneys, manual VOIs were placed in the renal parenchyma (renal cortex). For bone and muscle, irregular VOIs were placed in the 5th lumbar vertebra and the gluteal muscle accordingly. Due to its reasonably uniform tracer uptake, the liver parenchyma was used for background. Blood pool calculations were obtained from the average of the descending aorta VOI data, consisting of at least seven slices in sequential PET/CT images, placed centrally in the lumen of the aorta without including the aortic wall.

To quantitative compare SUV images and different K_i_ images (STD, STI and LTI), target-to-background ratio (TBR) and contrast-to-noise ratio (CNR) for individual tumor lesions were calculated, as described in Eqs. 1–4. The liver parenchyma was used as background for these calculations.

1 $$\:{\text{T}\text{B}\text{R}}_{\text{mean}}\:=\frac{\text{M}\text{e}\text{a}\text{n}\left({\text{V}\text{O}\text{I}}_{\text{L}\text{e}\text{s}\text{i}\text{o}\text{n}}\right)\:}{\text{M}\text{e}\text{a}\text{n}\left({\text{V}\text{O}\text{I}}_{\text{B}\text{a}\text{c}\text{k}\text{g}\text{r}\text{o}\text{u}\text{n}\text{d}}\right)\:}$$

2 $$\:{\text{C}\text{N}\text{R}}_{\text{mean}}\:=\frac{\text{M}\text{e}\text{a}\text{n}\left({\text{V}\text{O}\text{I}}_{\text{L}\text{e}\text{s}\text{i}\text{o}\text{n}}\right)\:-\:\text{M}\text{e}\text{a}\text{n}\left({\text{V}\text{O}\text{I}}_{\text{B}\text{a}\text{c}\text{k}\text{g}\text{r}\text{o}\text{u}\text{n}\text{d}}\right)\:}{\text{S}\text{T}\text{D}\left({\text{V}\text{O}\text{I}}_{\text{B}\text{a}\text{c}\text{k}\text{g}\text{r}\text{o}\text{u}\text{n}\text{d}}\right)\:}$$

3 $$\:{\text{T}\text{B}\text{R}}_{\text{max}}\:=\frac{\text{M}\text{a}\text{x}\left({\text{V}\text{O}\text{I}}_{\text{L}\text{e}\text{s}\text{i}\text{o}\text{n}}\right)\:}{\text{M}\text{e}\text{a}\text{n}\left({\text{V}\text{O}\text{I}}_{\text{B}\text{a}\text{c}\text{k}\text{g}\text{r}\text{o}\text{u}\text{n}\text{d}}\right)\:}$$

4 $$\:{\text{C}\text{N}\text{R}}_{\text{max}}\:=\frac{\text{M}\text{a}\text{x}\left({\text{V}\text{O}\text{I}}_{\text{L}\text{e}\text{s}\text{i}\text{o}\text{n}}\right)\:-\:\text{M}\text{e}\text{a}\text{n}\left({\text{V}\text{O}\text{I}}_{\text{B}\text{a}\text{c}\text{k}\text{g}\text{r}\text{o}\text{u}\text{n}\text{d}}\right)\:}{\text{S}\text{T}\text{D}\left({\text{V}\text{O}\text{I}}_{\text{B}\text{a}\text{c}\text{k}\text{g}\text{r}\text{o}\text{u}\text{n}\text{d}}\right)\:}$$

#### VOI-based evaluation of dynamic PET/CT data using 2 tissue-compartment model

Besides the Patlak model, we also used a VOI-based analysis based on a 2 tissue-compartment model (2TCM) to our dynamic data using PKIN module of PMOD software. Due to the complexity of the 2TCM and limited performance of the dedicated software, we only focused on the VOI-based evaluation of 2TCM and not on the calculation of parametric images. In order to simulate the voxelwise 2TCM parametric imaging as closely as possible, we only choose the first fit result of iterative fitting of 2TCM without further manual fitting and without any parameter value restrictions. First fit result is a series of fitting either reaching the max number of iterations or reaching the minimum change of fitting criterions like ChiSquare. In order to compare the Patlak method with the 2TCM, we fixed the k_4_ to zero (irreversible 2TCM). Semi-quantitative evaluations were performed based on SUV calculations 50–60 min after tracer injection (the average SUV of the last two frames of the dynamic PET acquisition) generated from the VOIs placed over tumor lesions and normal organs. In addition, a detailed quantitative evaluation of the pharmacokinetics of [^18^F]FDG derived from the entire 60-min dynamic PET acquisition in tumor lesions and normal organs mentioned above was performed using a reversible 2TCM. The 2TCM includes the plasma compartment (C_plasma_), the transported [^18^F]FDG in C_1_ and the phosphorylated [^18^F]FDG (FGD-6-P) concentration in C_2_ [8]. The 2TCM fitting of averaged time-activity curves (TACs) from VOIs of tumor lesions and normal organs leads to the extraction of the parameters vB (unitless), K_1_ (mL/ccm/min), k_2_ (min^− 1^), k_3_ (min^− 1^) and k_4_ (min^− 1^). In particular, vB is the blood volume fraction, K_1_ and k_2_ are the uptake and clearance rate constants, whereas k_3_ represents the phosphorylation by hexokinase and k_4_ the dephosphorylation. Furthermore, the global tracer influx K_i_ (mL/ccm/min) was calculated from the compartment data using the formula: $\:{\text{K}}_{\text{i}}=\:({\text{K}}_{1}\times\:{\text{k}}_{3})/({\text{k}}_{2}+{\text{k}}_{3})$. The TBR_mean_ of K_i_ and k_3_ of VOI-based 2TCM were also calculated using Eq. 1. However, $\:\text{M}\text{e}\text{a}\text{n}\left({\text{V}\text{O}\text{I}}_{\text{L}\text{e}\text{s}\text{i}\text{o}\text{n}}\right)\:$and $\:\text{M}\text{e}\text{a}\text{n}\left({\text{V}\text{O}\text{I}}_{\text{B}\text{a}\text{c}\text{k}\text{g}\text{r}\text{o}\text{u}\text{n}\text{d}}\right)\:$ were simulated using single value of K_i_ or k_3_ fitted from the averaged TAC of the tissue VOIs instead of the average value of K_i_ or k_3_ fitted from each voxel TAC in the VOIs.

### Statistical analysis

Continuous variables were expressed as mean ± standard deviation (SD). Image quality parameters TBR_mean_ and CNR_mean_ of Patlak images and sumSUV images were compared using Wilcoxon matched-pairs signed-rank test. Further, differences between kinetic parameters of tumor lesions and normal organs were evaluated using the Student’s t-test. Correlations between the kinetic parameters K_i_ from 2TCM and K_i_ from Patlak images were investigated using Spearman’s rank correlation analysis. Statistical significance was considered for p-values less than 0.05. Statistical analysis was performed with Stata/MP 14.2 (StataCorp LLC).

## Results

**Fig. 1:**
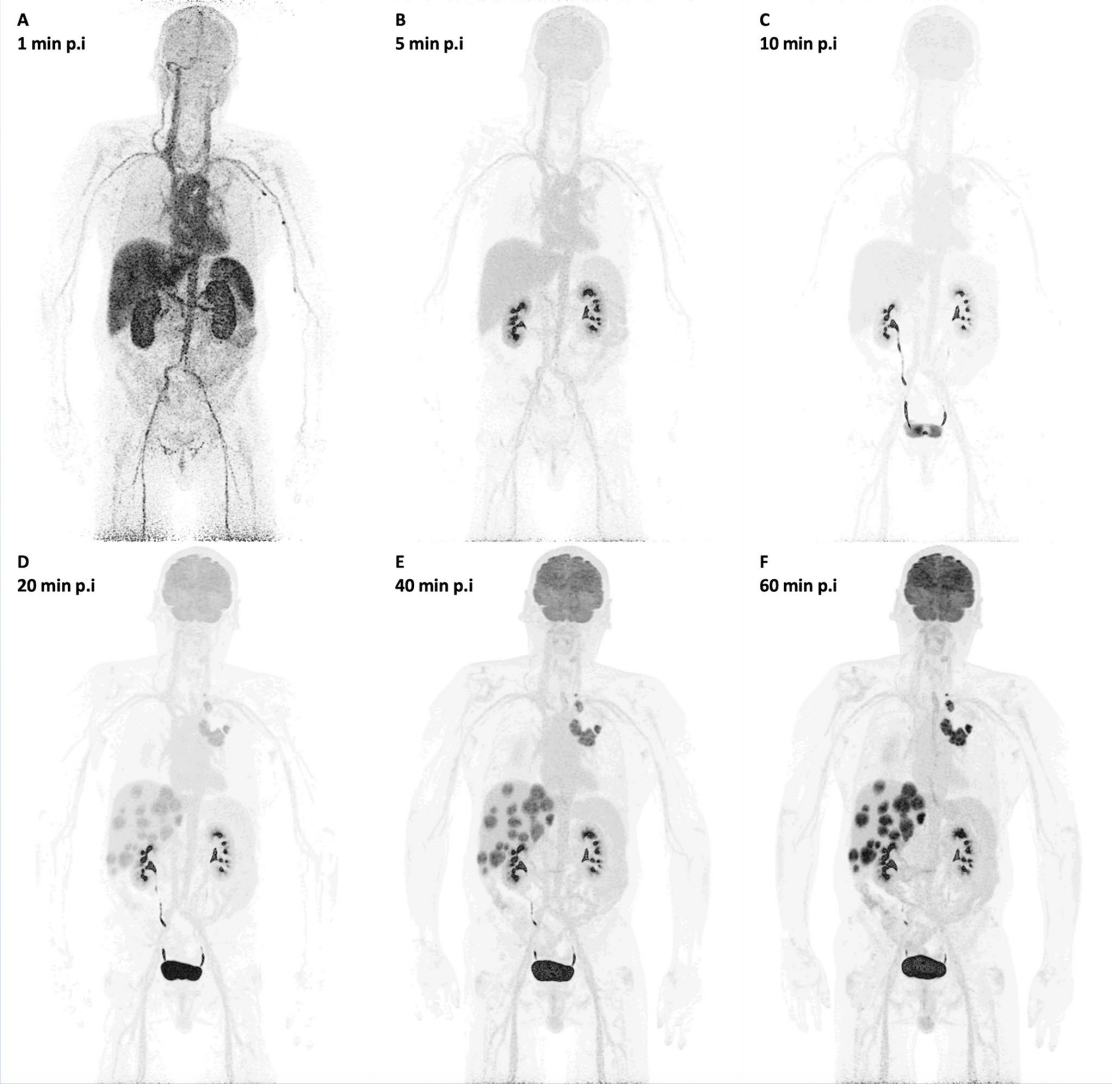
Example of whole-body dynamic images acquired at different time points of dynamic PET acquisition of a patient with primary lung tumor and multiple liver metastasis. The images shown depict acquisitions at 1 (**A**), 5 (**B**), 10 (**C**), 20 (**D**), 40 (**E**) and 60 min (**F**) after administration of [^18^F]FDG. The patient has multiple liver metastases from a primary lung tumor. Notably, some liver metastases can be delineated already 20 min after tracer injection

**Fig. 2:**
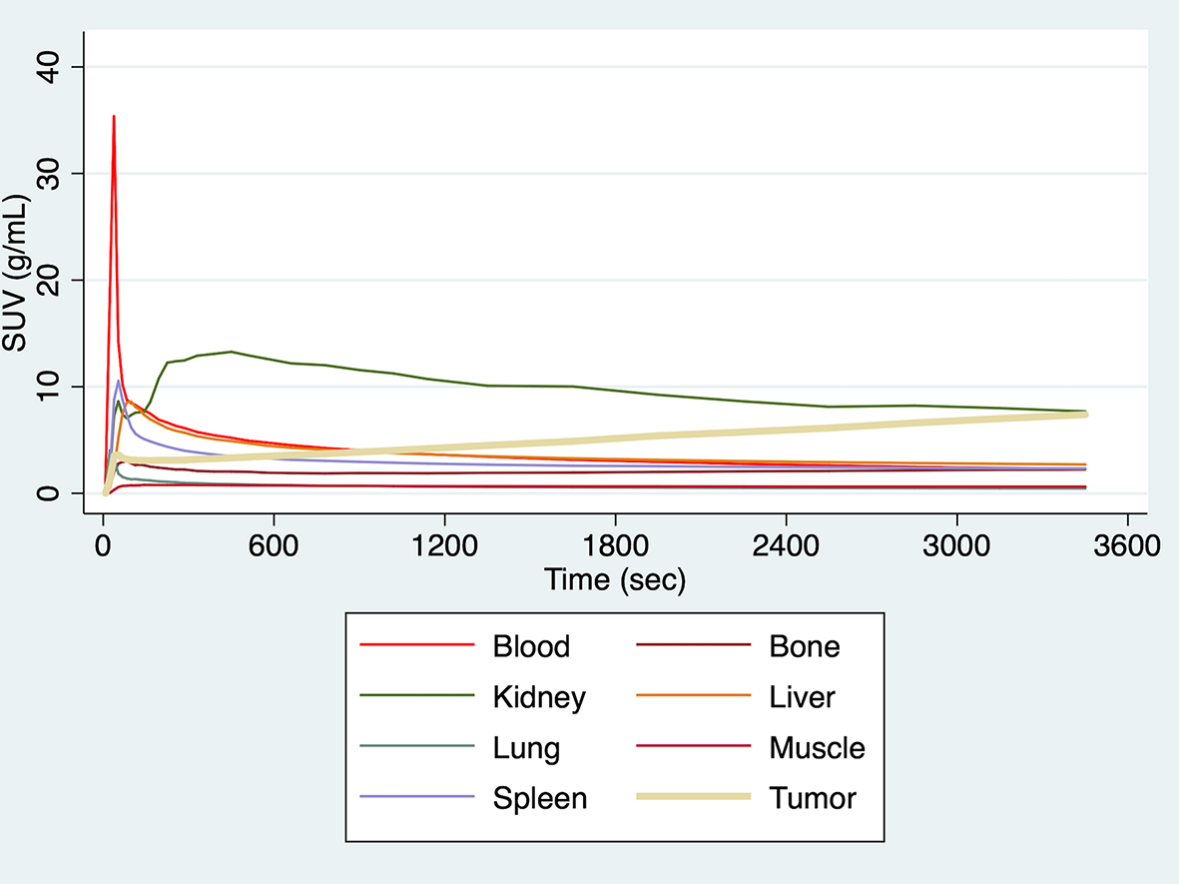
Time-activity curves (TACs) of [^18^F]FDG derived from whole-body dynamic PET/CT studies using a 33-frame protocol (y-axis, average SUV; x-axis, time in sec). The TACs represent the mean values of all evaluated VOIs corresponding to normal organs and tumor lesions. TACs of the blood pool (descending aorta), bone, kidney, liver, lung, muscle, spleen and tumor lesions are presented

An example of whole-body dynamic image acquired at different time points of dynamic PET acquisition of a patient with a primary lung tumor and liver metastases is provided in Fig. 1. Dynamic PET/CT scanning also led to the generation of average TACs in tumor lesions and reference tissue, which are presented in Fig. 2. In general, the curves derived from normal organs demonstrated a peak shortly after the tracer injection followed by a decreasing uptake over time except the kidney. In particular, the kidney showed a longer increase up to 8 min followed by a decreasing concentration over time. However, tumor lesions exhibited lower initial peaks and steadily increasing slope of the TACs.

### Visual assessment of different patlak parametric images and comparison with the sumSUV

**Fig. 3:**
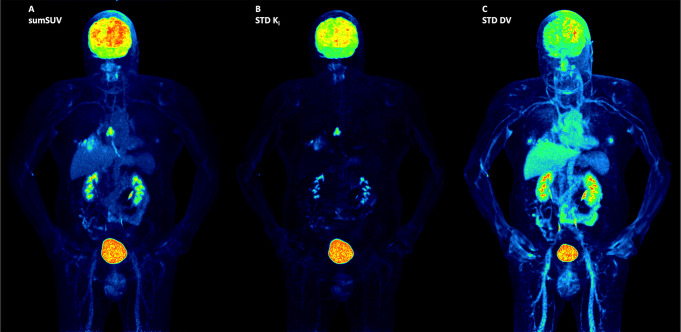
sumSUV, K_i_ and DV of STD Patlak MIP images from a lung cancer patient. The K_i_ images show a higher phosphorylation rate in the primary tumor in the right lung as compared to the radiation necrosis in the dorsal part of the right lung. DV images demonstrate a higher DV in the radiation necrosis than in the primary lung tumor

In terms of visual analysis, Patlak K_i_ images demonstrated much better contrast compared to sumSUV images. We observed less background for normal organs in Patlak K_i_ images compared to sumSUV. In a patient with a lung tumor in the right lung and radiation necrosis after radiotherapy, the parametric K_i_ images showed a higher phosphorylation rate in the lung tumor as compared with the radiation necrosis (Fig. 3). The parametric DV images demonstrate a low DV in the lung tumor and higher DV in the radiation necrosis.

**Fig. 4:**
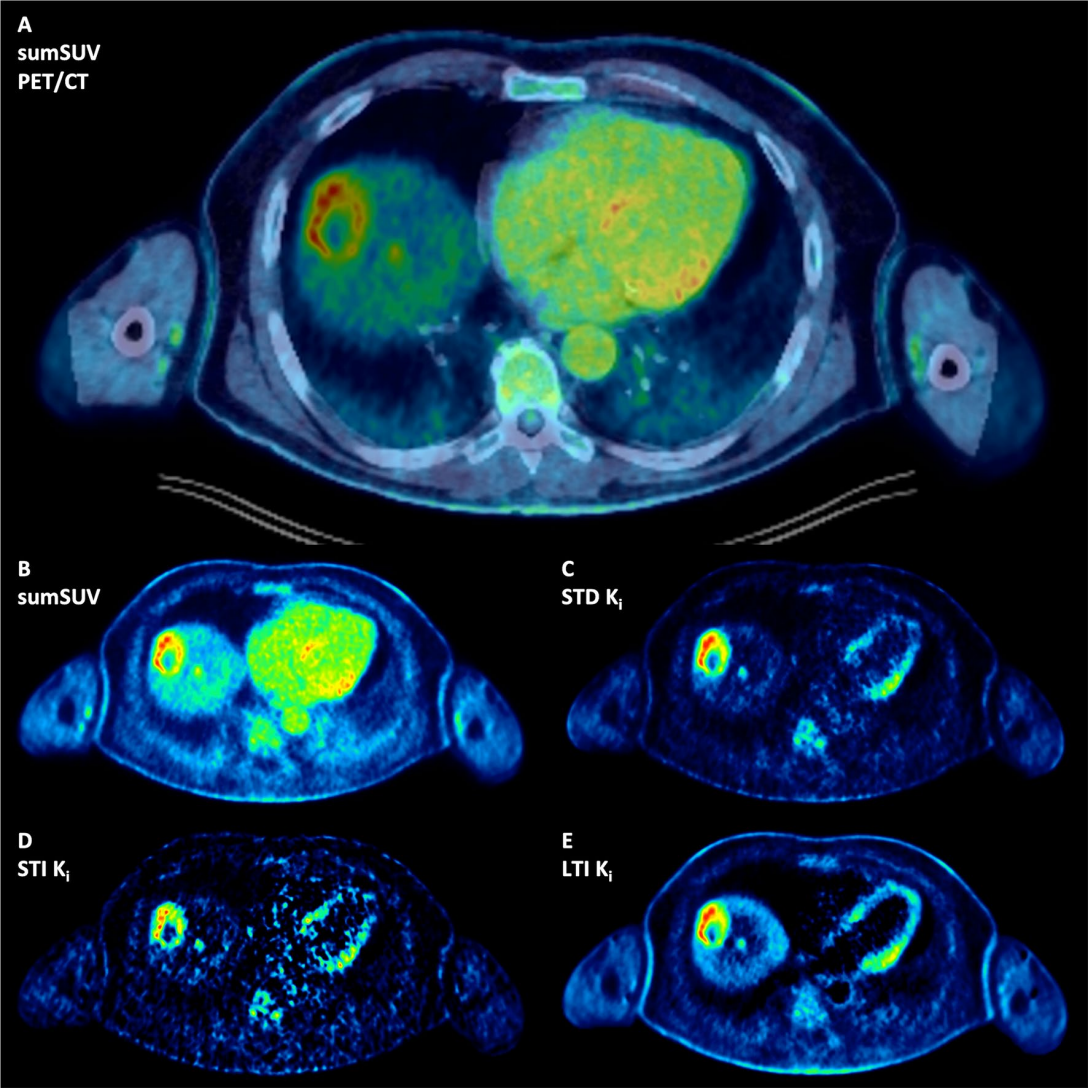
Patient with multiple liver metastases from uveal melanoma: All Patlak K_i_ images demonstrate a small liver metastasis in segment 8 which is not clearly delineated in the sumSUV (**A** fused PET-CT and **B**) SUV images due to high background of the liver. The STD Patlak K_i_ image (**C**) demonstrates better contrast and less background than LTI Patlak K_i_ image (**E**) and less noise than STI Patlak k_i_ image (**D**)

**Fig. 5:**
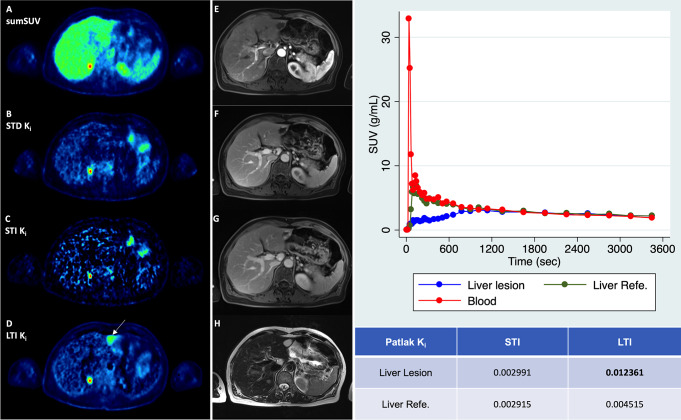
Another patient with multiple liver metastases from uveal melanoma. All images demonstrate a small liver metastasis in the segment 7 of the right liver lobe. Interestingly, only LTI Patlak k_i_ image (**D**) demonstrated an additional liver lesion in the left lobe. This is a case where Patlak revealed a lesion which could not be delineated by sumSUV image (**A**) due to the liver equivalent [^18^F]FDG uptake in the liver lesion. However, MRI revealed a hemangioma: MRI image of the arterial phase of a contrast-enhanced T1 Dixon sequence depicted a hypointense lesion with discrete peripheral enhancement (**E**), while contrast-enhancement progressed centripetally in the venous (**F**) and delayed images (**G**). H: T2-weighted images showed the hemangioma as typically T2-hyperintense. Time-activity curves (TACs) of blood, liver lesion and liver reference (y-axis, average SUV; x-axis, time in sec) are illustrated in the right part

Regarding the number of lesions, no significant differences were observed between the three Patlak approaches used (STD K_i_, STI K_i_ and LTI K_i_) with two positive findings, one true positive and one false positive, presented in Figs. 4 and 5. In Fig. 4 we compared sumSUV with STD, STI and LTI Patlak K_i_ images in a patient with liver metastases from uveal melanoma. In this case, all Patlak K_i_ images delineated a small liver metastasis in segment 8 which was not clearly visible on sumSUV images (A and B) due to high background. The STD Patlak K_i_ image (C) showed better contrast and less background than the LTI Patlak K_i_ image (E) and less noise than the STI Patlak K_i_ image (D). Another patient with multiple liver metastases from uveal melanoma is presented in Fig. 5, where LTI Patlak K_i_ images delineated one lesion in the left liver lobe, which was not visible in sumSUV due to the liver equivalent [^18^F]FDG uptake. Interestingly, this liver lesion was a hemangioma in MRI (Fig. 5E-H) and therefore, LTI K_i_ images were false positive. Figure 5 (right side) shows the time-activity curves (TACs) derived from this patient and provides an explanation for this finding only in LTI. The curves show that the liver lesion has a different TAC from the beginning but similar TAC from around 1200 s with the normal surrounding liver tissue (reference). Therefore, it not possible to differentiate them according to the short-time Patlak method. In STI Patlak, the K_i_ value of the tumor tissue is 0.002991 which is comparable to 0.002915 of the normal tissue. However, in LTI Patlak, the K_i_ value (0.012361) of the tumor tissue is much higher than of the normal tissue (0.004515).

**Fig. 6:**
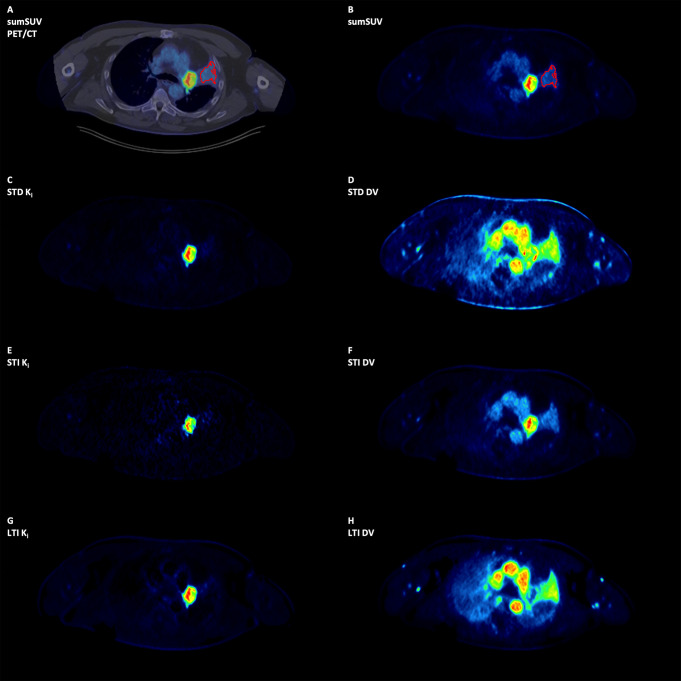
Patient with a primary lung tumor and atelectasis. The Patlak images are true negative compared to the sumSUV image. In the sumSUV (**A** fused PET-CT and **B**) images there is [^18^F]FDG enhancement in the atelectasis, but not in any of the Patlak K_i_ images (**C**, **E**, **G**). All DV images (**D**, **F**, **H**) showed enhanced contrast in the atelectasis and variable contrast in the primary tumor depending on the method used. In particular, LTI DV images (**H**) showed a rim-like uptake in the primary lung tumor. The DV enhancement in the atelectasis is related to higher perfusion-related [^18^F]FDG transport but not phosphorylation, explaining the enhanced SUV

We observed also one true negative finding on Patlak images as compared with sumSUV in a patient with a lung tumor and atelectasis (Fig. 6). In the sumSUV images (A fused PET-CT and B) there is a low [^18^F]FDG enhancement in the atelectasis, but not in any of the Patlak K_i_ images (C, E, G). All DV images (D, F, H) showed enhanced contrast in the atelectasis and variable contrast in the primary tumor depending on the method used. In particular, LTI DV images (H) showed a rim-like uptake in the primary lung tumor. The enhanced [^18^F]FDG uptake in the atelectasis of the SUV images was due to the enhanced [^18^F]FDG transport as demonstrated in the DV images (Fig. 6, right side).

### Objective evaluation of image quality and comparison between different patlak images and sumSUV images

**Fig. 7:**
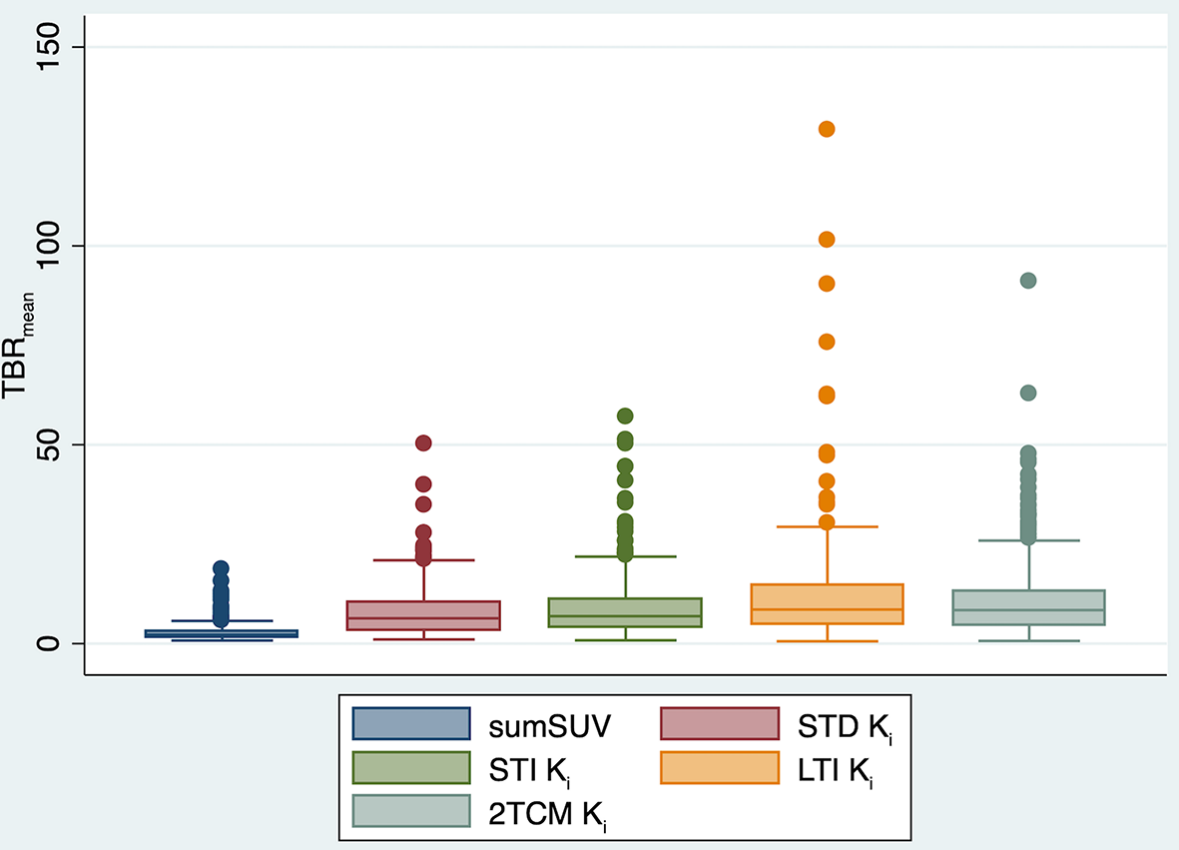
Boxplots of TBR_mean_ for sumSUV, STD K_i_, STI K_i_, LTI K_i_ and 2TCM K_i_

TBR and CNR values for 346 tumor lesions summarized in Table 2 confirmed the general findings mentioned above. Patlak images of STD, STI and LTI were superior to sumSUV images in all evaluations, showing 2.9-, 3.3- and 4.3-fold higher TBR_mean_ as compared with corresponding sumSUV images accordingly. Regarding noise, due to the limited time frame, the STI K_i_ images demonstrated the worst CNR_mean_, showing 0.73 fold lower TBR_mean_ as compared with corresponding sumSUV images. The STD K_i_ images revealed similar CNR_mean_ as compared with the LTI K_i_ images, demonstrating that the direct Patlak method can handle the noise problem based on a shorter acquisition time to generate parametric imaging. The TBR_mean_ of k_3_ and K_i_ from 2TCM was also assessed and will be discussed in the next section. The boxplots of TBR_mean_ for sumSUV, K_i_ of different Patlak methods and 2TCM are illustrated in Fig. 7.

**Table 2:** Descriptive statistics of TBR and CNR of sumSUV, different Patlak K_i_ imaging and VOI-based 2TCM parameters. Please note that normal liver parenchyma was used as background

		Patlak			2TCM	
	sumSUV	STD K_i_	STI K_i_	LTI K_i_	K_i_	k_3_
TBR_mean_	2.78 ± 2.07	8.08 ± 6.38	9.25 ± 8.09	11.94 ± 13.31	9.86 ± 8.97	58.67 ± 119.80
TBR_max_	4.48 ± 3.82	14.43 ± 12.08	18.45 ± 17.81	19.70 ± 22.93	*	*
CNR_mean_	22.73 ± 28.86	29.21 ± 27.12	12.95 ± 13.85	29.64 ± 29.62	*	*
CNR_max_	44.23 ± 53.09	54.97 ± 49.92	27.25 ± 30.13	51.15 ± 52.54	*	*

*No max and SD available for VOI-based 2TCM

The results of the Wilcoxon matched-pairs signed-rank test, presented in Table 3, showed the differences in TBR and CNR between the four image sets. The best result was demonstrated for the LTI K_i_ image. The superiority of TBR_mean_ in LTI Patlak K_i_ images as compared with sumSUV images was evident in 345/346 lesions. The comparison of STD with LTI demonstrated that the TBR_mean_ of LTI Patlak K_i_ was higher in 306/346 of tumor lesions. TBR_mean_ of STI Patlak K_i_ was higher than STD Patlak Ki in only 225/346 of tumor lesions.

**Table 3:** The positive rank number of Wilcoxon signed-rank for four sets of TBR_mean_ and CNR_mean_. The number of tumor tissue is 346. Four sets of TBR_mean_ are significantly different to each other. Four sets of CNR_mean_ are significantly different to each other except LTI Patlak K_i_ with STD Patlak K_i_

Positive Rank of TBR_mean_ / CNR_mean_	LTI Patlak K_i_	STI Patlak K_i_	STD Patlak K_i_	sumSUV
LTI Patlak K_**i**_	1			
STI Patlak K_**i**_	248*/331*	1		
STD Patlak K_**i**_	306*/187	225*/21**	1	
sumSUV	345*/313*	345*/63**	342*/287*	1

* Row label is significantly higher than the column label (*p* = 0.0000) ** Row label is significantly lower than the column label (*p* = 0.0000)

### Comparison of kinetic parameters between VOI based 2TCM and patlak model for normal tissue and tumor lesions

Three hundred and forty six (346) [^18^F]FDG-positive tumor lesions, as well as normal organs, including the liver, kidney, spleen, lung, muscle and bone, were evaluated both semi-quantitatively and quantitatively by means of dynamic PET/CT. The results of the semi-quantitative and quantitative evaluation are presented in Table 4.

**Table 4:** Descriptive statistics of dynamic PET data of [^18^F]FDG in normal organs and tumor lesions. SUV refers to the average uptake value calculated from the dynamic acquisitions performed 50–60 min after injection. The kinetic parameters of 2TCM were calculated from the entire 60-min dynamic acquisition. Patlak K_i_ parameters were the average values of the VOIs from the K_i_ parametric images. All parameters were compared using t-test between normal organs and tumor lesions. All significant results (*p* < 0.05) are tagged with * or **

		2TCM					Patlak		
	sumSUV	vB	K_1_	k_2_	k_3_	K_i_	STD K_i_	STI K_i_	LTI K_i_
Liver	3.386 ± 1.203**	0.002 ± 0.017**	0.571 ± 0.154*	0.674 ± 0.185**	0.005 ± 0.004**	0.004 ± 0.002**	3.50e-06 ± 1.65e-06 **	0.0061 ± 0.0026**	0.0035 ± 0.0024**
Kidney	9.612 ± 8.361	0.066 ± 0.075	0.594 ± 0.427*	0.428 ± 0.611**	0.013 ± 0.034**	0.012 ± 0.020**	1.18e-05 ± 1.03e-05**	0.0263 ± 0.0240**	0.0278 ± 0.0417
Spleen	2.901 ± 1.024**	0.028 ± 0.045**	1.478 ± 0.661*	2.553 ± 1.087*	0.010 ± 0.005**	0.005 ± 0.002**	4.12e-06 ± 1.63e-06**	0.0067 ± 0.0032**	0.0039 ± 0.0020**
Lung	0.579 ± 0.442**	0.120 ± 0.053*	0.014 ± 0.016**	0.262 ± 0.250**	0.159 ± 0.414	0.0004 ± 0.0004**	5.89e-07 ± 3.07e-07**	0.0010 ± 0.0009**	0.0005 ± 0.0003**
Muscle	0.775 ± 0.255**	0.0003 ± 0.001**	0.051 ± 0.031**	0.416 ± 0.275**	0.015 ± 0.005**	0.0018 ± 0.0006**	1.39e-06 ± 4.23e-07**	0.0021 ± 0.0010**	0.0021 ± 0.0009**
Bone	2.7723 ± 1.265**	0.007 ± 0.013**	0.248 ± 0.108	0.888 ± 0.420	0.028 ± 0.009**	0.008 ± 0.003**	6.44e-06 ± 2.24e-06**	0.0106 ± 0.0038**	0.0077 ± 0.0025**
Tumor lesions	8.892 ± 7.242	0.057 ± 0.090	0.267 ± 0.249	0.863 ± 0.809	0.234 ± 0.425	0.033 ± 0.026	2.67e-05 ± 2.32e-05	0.0579 ± 0.0585	0.0344 ± 0.0273

* Significantly lower values for tumor lesions than normal organs (*p* < 0.05) ** Significantly higher value for tumor lesions than normal organs (*p* < 0.05)

**Fig. 8:**
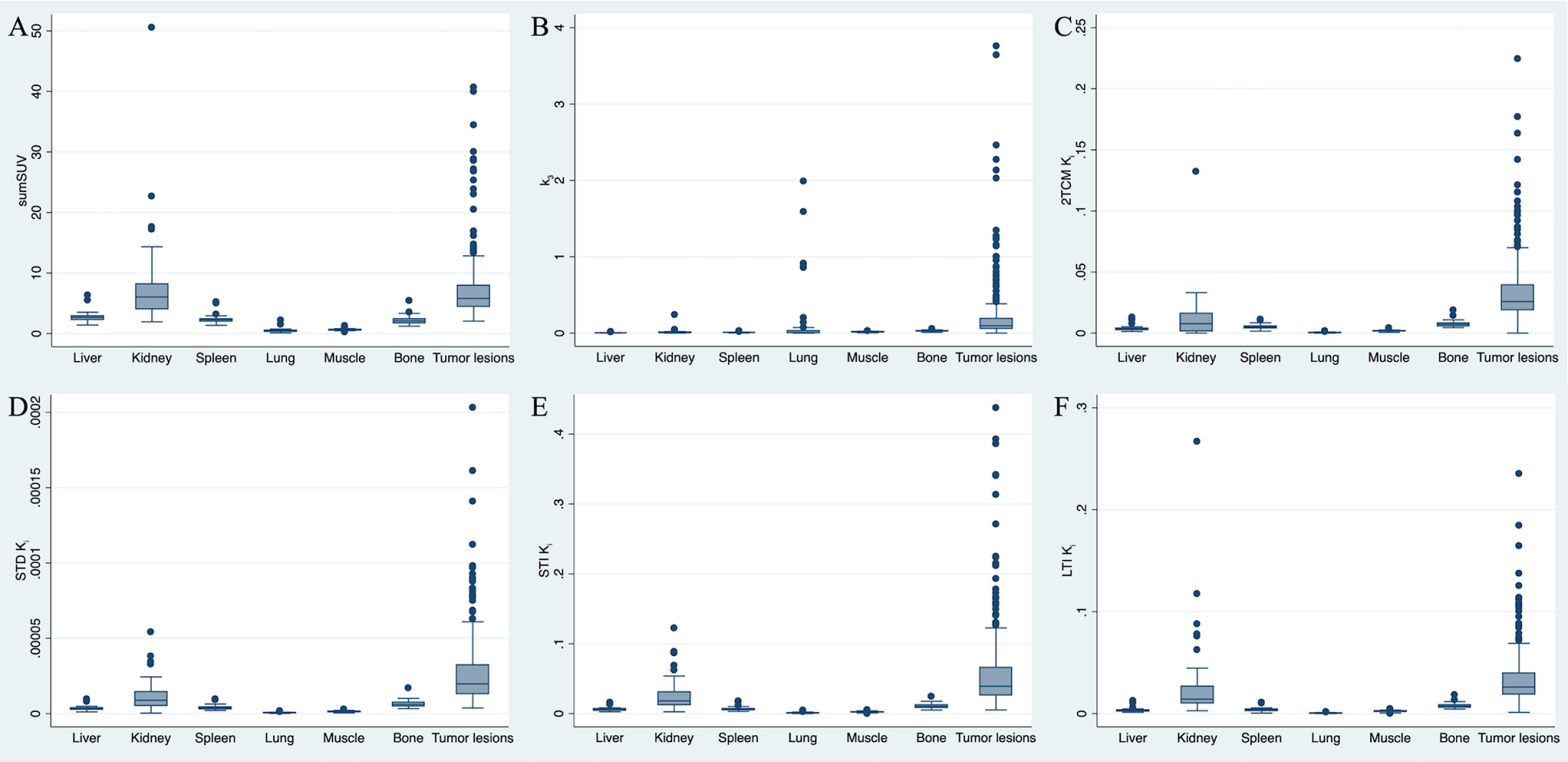
Boxplots of sumSUV, k3 and K_i_ of 2TCM and K_i_ of Patlak models (STD, STI and LTI) grouped by different normal tissues and tumor lesions

Briefly, sumSUV was significantly higher for tumor lesions compared to all normal organs except kidney. For 2TCM, Table 4 shows that k_3_ and K_i_ revealed the most significantly higher values between tumor lesions and normal tissues. In particular, k_3_ was significantly higher in all evaluated tumor lesions except normal lung, whereas K_i_ was significantly higher in all evaluated tumor lesions. Regarding the Patlak model, K_i_ values demonstrated significantly higher values in tumors as compared with normal tissues for each Patlak approach (STD, STI, LTI) except for the kidney when using the LTI approach. The boxplots of sumSUV, k_3_ and K_i_ of 2TCM and K_i_ of Patlak models (STD, STI and LTI) grouped by different normal tissues and tumor lesions are illustrated in Fig. 8.

The TBR_mean_ of 2TCM K_i_ and k_3_ are also demonstrated in Table 2. In particular, K_i_ of 2TCM has similar TBR_mean_ values (9.86) to the K_i_ of the Patlak (8.08–11.94). In contrast, 2TCM k_3_ showed the highest TBR_mean_ values (58.67) as compared to all other K_i_ parameters (STD, STI, LTI, 2TCM) and was almost 21-fold higher than sumSUV. As mentioned in the materials and methods section, liver parenchyma was used as background for the calculation of TBR. This means that 2TCM k_3_ has the highest discriminatory ability between tumor lesion and normal liver tissue.

Table 5 shows the correlations between K_i_ parameters as calculated with all approaches (STD, STI, LTI, 2TCM) and for three different tissue groups, namely: all tissues, tumor lesions, normal tissues. Correlation analysis showed a very high statistically significant correlation (*p* = 0.00) between each K_i_, including K_i_ from 2TCM and K_i_ from Patlak with different protocols (STD, STI, LTI). In particular, we focused on the correlations between 2TCM K_i_ and parametric Patlak K_i_ values. 2TCM K_i_ revealed the highest correlation (*r* = 0.95) with the LTI Patlak K_i_ in tumor lesions group and demonstrated the highest correlation with the STD Patlak K_i_ in all tissues group and normal tissues group (*r* = 0.93 and *r* = 0.74 respectively).

**Table 5:** Spearman’s rank correlation test result of four sets of Ki in different groups of tissues. 2TCM K_i_ revealed the highest correlation (*r* = 0.95) with the LTI Patlak K_i_ in tumor lesions group and demonstrated the highest correlation with the STD Patlak K_i_ in all tissues group and normal tissues group (*r* = 0.93 and 0.74 respectively)

Rho (All tissues/ Tumor lesions/ Normal tissues)	2TCM K_i_	STD Patlak K_i_	STI Patlak K_i_	LTI Patlak K_i_
2TCM K_i_	1			
STD Patlak K_i_	0.9226*^#^/ 0.9232*/0.7146*^#^	1		
STI Patlak K_i_	0.8898*/0.8182*/0.6777*	0.9693*/0.9118*/0.9342*	1	
LTI Patlak K_i_	0.9058*/0.9529*^#^/0.7000*	0.9600*/0.9698*/0.8760*	0.9602*/0.8951*/ 0.9105*	1

* Significantly correlated (*p* = 0.0000) ^#^ The highest correlation between 2TCM K_i_ and Patlak K_i_

## Discussion

LAFOV and total body PET/CT systems have opened up new possibilities, particularly in oncological imaging, due to their higher sensitivity, the ability to perform WB imaging in a short time and due to the fact that these systems allow WB dynamic studies and therefore WB parametric imaging. This aspect is of particular importance for the assessment of pharmacokinetics of various, especially novel, radiopharmaceuticals [5]. Another aspect is the question if dynamic imaging can be introduced into clinical routine. This will be only the case if dynamic scanning and parametric imaging provide additional findings which have a clinical impact, e.g., a change in patient staging by up- or downstaging or a therapeutic decision.

Parametric imaging is a method of feature extraction method that allows the visualization of an isolated parameter of tracer kinetics based on dedicated mathematical models and a voxel-wise calculation. The advantage over a VOI-based pharmacokinetic analysis is the direct visualization of different kinetic parameters, such as tracer influx or transport rates (K_1_, k_2_, etc.), instead of calculating absolute numbers [12]. Specifically for [^18^F]FDG, Patlak imaging generates two parametric images, the so-called Influx or K_i_ images, which are related to the phosphorylated [^18^F]FDG, and the distribution volume or DV images, which are related to the perfusion-dependent and transported but not metabolized [^18^F]FDG.

The published data focusing on [^18^F]FDG demonstrate higher contrast of the parametric Patlak images compared to the summed [^18^F]FDG images approximately one hour after tracer injection, but do not show more findings on either on a patient or lesion basis. Fahrni et al. evaluated 18 oncological patients with different tumor entities and compared SUV to Patlak K_i_ images [14]. The authors also demonstrated a higher TBR and CNR for K_i_ as compared to SUV, which is comparable to our results. Overall, the results from 40 proven malignant lesions suggested a slightly improved sensitivity (from 92.5 to 95%) and accuracy (from 90.24 to 95.12%), and potentially improved specificity with K_i_ over SUV imaging. One lesion, later confirmed to be benign, was positive on SUV and negative on K_i_ .

Dias et al. investigated the impact of direct Patlak imaging in 109 oncological tumor patients with a Standard-Axial-Field-of-View (SAFOV) digital PET system of 26 cm FOV by using a multibed protocol [15]. The authors could not find any significant differences in the number of pathological lesions detected by direct Patlak as compared to conventional static images. However, they reported a higher TBR and CNR ratio for K_i_ images as compared to SUV. These results are comparable with our findings. Furthermore, they reported on 4 fewer false positive findings in Patlak K_i_ than in the SUV images. In our study, we observed three discordant findings in Patlak images as compared to sumSUV including one true positive, one false positive and one true negative.

Most of the published work is based on so-called postprocessing parametric Patlak images, which are based on the reconstructed PET images. However, software tools are available from manufacturers that allow the so-called direct image reconstruction of Patlak images based on the sinograms obtained [16–18]. This approach produces two sets of images, the distribution volume (DV) images, which reflect the perfusion-related part of [^18^F]FDG, and the influx or K_i_ images, which reflect the phosphorylated part of the tracer.

Sari et al. used both direct and indirect Patlak imaging in 24 oncological patients (49 tumor lesions) studied with [^18^F]FDG and LAFOV PET-CT (Biograph Vision Quadra) [8]. The authors reported that both direct and indirect Patlak imaging demonstrated superior TBR as compared with static SUV images. Regarding CNR, they reported a twofold higher CNR for direct than for indirect Patlak in tumor lesions, which is concordant with our results using the short-time protocols. There are some differences between the work of Sari et al. and our data. Firstly, Sari et al. did not use different time intervals for direct and indirect Patlak calculations. Secondly, the authors used the surrounding tissue and not the normal liver parenchyma as background tissue. The choice of background tissue is controversial and there is no general recommendation on the reference tissue, which should be used. However, the liver is used as reference in several papers and most importantly as a criterion for defining response to therapy with different response criteria, e.g. the Deauville criteria [19] for lymphoma and also in all sets of PERCIST criteria [20] .Other groups working with the Biograph Vision Quadra have also recently used the liver as a background [21]. We decided to use the normal liver parenchyma because it is clinically used also within different response criteria. We could demonstrate that TBR for LTI Patlak K_i_ were higher than for STD Patlak K_i_ and CNR for LTI Patlak K_i_ was comparable with CNR for STD Patlak K_i_ (Table 2). Furthermore, the authors compared the relationship between SUV and K_i_ values. They report a very strong correlation between SUV values and MRFDG values estimated using the direct Patlak method (*r* = 0.96) and the indirect Patlak method (*r* = 0.94). We focused on the correlation between K_i_ of 2TCM and Patlak. We found the highest correlation between the K_i_ of the VOI-based 2TCM with the K_i_ of LTI Patlak (*r* = 0.95) in tumor lesions group and the highest correlation with the STD Patlak K_i_ in all tissues group and normal tissues group (*r* = 0.93 and *r* = 0.74 respectively). We would also like to emphasize that our results are based on a larger cohort of 50 patients and 346 tumor lesions.

Wu et al. evaluated the impact of the time used for Patlak imaging in 65 patients with [^18^F]FDG and a total body PET-CT scanner (uExplorer) [22]. In this paper a voxelwise Patlak analysis was applied to generate K_i_ images based on IDIF as well as on a population -based- input function (PBIF) with different acquisition times (20–60, 30–60, 40–60, and 44–60 min) and found that the K_i_ images generated by the PBIF-based Patlak model using a 20-min dynamic scan achieved a similar diagnostic efficiency to images with IDIF from 40-min dynamic data. We used an IDIF in the descending aorta on at least 7 sequential images to create a VOI and did not use a PBIF. Therefore, we cannot compare our results to the work of Wu et al. concerning the impact of input function. Our goal was not to shorten the acquisition time but primarily to find the best time window for high quality Patlak images regarding TBR and CNR. Also Wu et al. reported on a better image quality, image noise and lesion conspicuity for longer time series than for shorter times. A limitation of the work of Wu et al. is that they did not focus on oncological patients but used [^18^F]FDG studies in different patients not further specified or in volunteers.

Overall, the selection of the time frame has an impact on the Patlak analysis. This means that even for a simple parametric method like Patlak imaging, there are still some variables that need to be adjusted. From this point of view, the indirect Patlak method has an advantage because the equilibrium time can be easily and quickly adjusted. As there is no generally accepted threshold for the equilibrium time, we wanted to compare the standard approach and assess the potential benefits of a long time protocol. The results demonstrate that the contrast is higher for the long time protocols. The selection of the appropriate time frame for Patlak analysis is crucial. Our original idea was to compare the standard Patlak approach using the last 30 min of the 60 min acquisition with a long time protocol consisting of 59 min by excluding only the initial part of the curve with the peak of the tracer uptake. Additionally to the exclusion of the first 45 s we calculated also a long time protocol by excluding the first 300 s. The 55Min-LTI results were comparable to the 59Min-LTI results, which are presented in the supplement.

The impact of the applied dose with this LAFOV system has been investigated in our previous works [23, 24]. The standard dose for [^18^F]FDG in Germany is 3 MBq/kg. We decided to use 2 MBq/kg in order to reduce the radiation exposure in patients, taking also into consideration that most of them have been studied longitudinally. In terms of image quality it would have been probably better not to reduce the radiopharmaceutical dose, but this was considered a reasonable compromise for performing dynamic [^18^F]FDG PET/CT studies in oncological patients combined with a standard static PET/CT protocol, which is necessary for clinical purposes.

In most published papers, researchers either used VOI-based pharmacokinetic modeling depending on the tracer, such as 1TCM or 2TCM, or they used mostly parametric Patlak imaging to differentiate between normal tissue and tumor lesions, but not the combination of both [23, 25–27], with only few exceptions [28]. In this work, we performed a combined evaluation and observed that different models have different performance between normal tissues and tumor lesions. In particular, we found that K_i_ from Patlak imaging performed better in normal lung but k_3_ based on 2TCM performed better in normal liver. Therefore, a combination of the different models and AI-based approaches that allow for a better image segmentation of normal organs as well as improved compartment modelling may lead to better results and help to calculate parametric images with better TBR in the whole body [29, 30].

### Limitations

There are some limitations in our work. Firstly, we did not evaluate the long time Patlak using the direct approach for technical reasons. Another limitation is that it would be probably preferable to compare Patlak modeling with 2TCM voxel-wise parametric imaging. Patlak imaging is a linear approach and not comparable to the more complex iterative fitting based 2TCM method. However, a general problem and limitation is that 2TCM is based on several assumptions and is operator dependent, depending for example on the type of input function, delay correction, local minimum of iterative fitting, fixed parameters such as VB and k4 etc. Some of these problems can be handled manually for VOI-based approaches, but cannot be easily solved for voxel-based parametric imaging. Therefore, in the current work we couldn’t assess 2TCM parametric imaging and compare it directly with Patlak imaging. However, we will soon have access to the appropriate software for such evaluations and will make this comparison in a future work [31]. A technical limitation is the fact that we could not use more frames for the reconstruction of a dynamic data acquisition due to the large data volume. This may have an impact on short time indirect Patlak calculations (STI). However, this fact does not affect direct Patlak calculations, which are based on sinograms. Finally, not all metastatic findings were histologically confirmed. However, all patients had a histologically confirmed primary tumor and at least one metastatic lesion prior to treatment. It is well known that it is impossible to have a histologic confirmation of every lesion. Moreover, all patients had additional imaging with either diagnostic, contrast-enhanced CT or MRI. For example, we managed to further assess the questionable finding in LTI Patlak K_i_ image by additional liver MRI.

## Conclusion

Dynamic [^18^F]-FDG PET/CT is feasible with the new LAFOV PET/CT scanner and produces Patlak K_i_ images of good visual quality and better lesion contrast than SUV images, regardless of the Patlak methods used (direct or indirect, short-time or long-time). In few cases, Patlak images revealed discordant findings as compared with sumSUV and added further useful clinical information. However, Patlak imaging does not increase lesion detection rate as compared with the sumSUV images. In addition, the TBR of LTI Patlak K_i_ images is significantly higher than that of STD Patlak K_i_ images. Our results demonstrate, that the time window used for Patlak reconstruction plays a more important role than the use of (in)direct image reconstruction. Different model parameters vary in their differential capability between tumor lesion and normal tissue. In particular, Patlak K_i_ works better in normal lung and k_3_ of 2TCM works better in normal liver. Compared to 2TCM, the Patlak imaging is simple, stable and in particular the indirect Patlak method is fast. A combination of different models, such as Patlak and 2TCM may be helpful in parametric imaging to get the best TBR in the whole body in future studies.

## Electronic supplementary material

Below is the link to the electronic supplementary material.

Supplementary Material 1

## Data Availability

The datasets generated during and/or analysed during the current study are available from the corresponding author on reasonable request.
