# Supplementary material for: Impact of different parametric Patlak imaging approaches and comparison with a 2-tissue compartment pharmacokinetic model with a long axial field-of-view (LAFOV) PET/CT in oncological patients
Source: Eur J Nucl Med Mol Imaging. 2024 Sep 11;52(2):623–37. doi: 10.1007/s00259-024-06879-4 (PMC11732916; doi:10.1007/s00259-024-06879-4)
Supplement: Supplementary file 1 — Supplementary Material 1 [file 259_2024_6879_MOESM1_ESM.docx]

|  |  | **Patlak** | | | | **2TCM** | |
| --- | --- | --- | --- | --- | --- | --- | --- |
|  | **sumSUV** | **STD K_i_** | **STI K_i_** | **LTI-55Min K_i_** | **LTI-59Min K_i_** | **K_i_** | **k_3_** |
| TBR_mean_ | 2.78±2.07 | 8.08±6.38 | 9.25±8.09 | 11.07±9.87 | 11.94±13.31 | 11.07 ± 10.31 | 68.90 ± 142.68 |
| TBR_max_ | 4.48±3.82 | 14.43±12.08 | 18.45±17.81 | 18.73±17.82 | 19.70±22.93 | * | * |
| CNR_mean_ | 22.73±28.86 | 29.21±27.12 | 12.95±13.85 | 27.26±27.58 | 29.64±29.62 | * | * |
| CNR_max_ | 44.23±53.09 | 54.97±49.92 | 27.25±30.13 | 48.08±49.73 | 51.15±52.54 | * | * |

**Table S1** Descriptive statistics of TBR and CNR of sumSUV, different Patlak K_i_ imaging and VOI-based 2TCM parameters. Please note that normal liver parenchyma was used as background. Considering the equilibrium time, the long-time-indirect Patlak K_i_ imaging has been calculated also with 55 minutes, which skipped the first 300 seconds.

**Table S2** Descriptive statistics of fit quality parameters of 2TCM in normal organs and tumor lesions.

|  | 2TCM | | |
| --- | --- | --- | --- |
|  | ChiSquare | AIC | MSC |
| Liver | 12.407 ± 4.684 | 85.928 ± 12.835 | 1.916 ± 0.384 |
| Kidney | 27.141 ± 33.302 | 95.831 ± 33.811 | 1.366 ± 0.509 |
| Spleen | 10.271 ± 9.601 | 68.352 ± 30.470 | 2.865 ± 0.845 |
| Lung | 103.811 ± 121.240 | 139.426 ± 36.330 | 1.441 ± 1.018 |
| Muscle | 8.858 ± 5.256 | 70.899 ± 21.505 | 1.715 ± 0.605 |
| Bone | 6.188 ± 3.931 | 59.145 ± 20.609 | 1.948 ± 0.607 |
| Tumor lesions | 16.460 ± 35.907 | 62.059± 46.659 | 2.518± 1.342 |
